# Hole-mask colloidal nanolithography combined with tilted-angle-rotation evaporation: A versatile method for fabrication of low-cost and large-area complex plasmonic nanostructures and metamaterials

**DOI:** 10.3762/bjnano.5.68

**Published:** 2014-05-06

**Authors:** Jun Zhao, Bettina Frank, Frank Neubrech, Chunjie Zhang, Paul V Braun, Harald Giessen

**Affiliations:** 1 4. Physics Institute and SCoPE Research Center, University of Stuttgart, Pfaffenwaldring 57, 70569 Stuttgart, Germany; 2 Department of Materials Science and Engineering, University of Illinois at Urbana Champaign, 312E MSEB, MC-246, 1304 W. Green Street, Urbana, Illinois 61801, United States

**Keywords:** hole-mask colloidal nanolithography, localized surface plasmon resonance sensing, low-cost large-area plasmonic nanostructures, multilayer fabrication, surface-enhanced infrared absorption spectroscopy (SERS)

## Abstract

Many nano-optical applications require a suitable nanofabrication technology. Hole-mask colloidal nanolithography has proven to be a low-cost and large-area alternative for the fabrication of complex plasmonic nanostructures as well as metamaterials. In this paper, we describe the fabrication process step by step. We manufacture a variety of different plasmonic structures ranging from simple nano-antennas over complex chiral structures to stacked composite materials for applications such as sensing. Additionally, we give details on the control of the nanostructure lateral density which allows for the multilayer-fabrication of complex nanostructures. In two accompanying movies, the fabrication strategy is explained and details are being demonstrated in the lab. The movies can be found at the website of Beilstein TV.

## Introduction

Optics with metallic nanostructures has generated keen interest over the last few years. The resonant excitation of particle plasmons and their ability to concentrate light on subwavelength scales has led to a plethora of fundamental investigations. Initially, investigations on the tuning of single and simple plasmonic nanostructures were carried out, which demonstrated the possibility to tune particle plasmon resonances by varying geometries, materials, and substrates. Recently, more complex geometries such as dimers, oligomers, stacked and chiral structures, and hybrid combinations with different materials were examined [1–3]. Mode coupling, plasmon hybridization, and the interaction of bright and dark modes, which leads to plasmonic Fano resonances, were discussed [4–8]. The structure geometries went from simple planar dot-like structures to complex, hybrid 3-dimensional (3D) systems. First applications in the area of sensing in the visible and near-infrared spectral range as well as antenna-assisted surface-enhanced infrared absorption (SEIRA) were demonstrated [9–10]. Also, nano-antennas with their ability to enhance light emission from single emitters as well as to concentrate incoming light into hot spots were utilized. Metamaterials based on metallic split-ring resonators (SRRs) were able to simultaneously demonstrate negative dielectric permittivity as well as magnetic permeability, which leads to a negative refractive index [11]. In hybrid solar cells and organic light emitting diodes, plasmonic nanostructures enhanced the efficiency. The combination of plasmonic nanostructures and semiconductors led to surface plasmon lasers (SPASERs) [12–13]. Hybrid materials that combined magnetooptical layers with plasmonic nanostructures enhanced the Faraday and magneto-optical Kerr effect. Tailored nanostructures were able to lead to surface-enhanced Raman scattering (SERS), in particular when the particle plasmon resonance was tuned to the pump laser wavelength. Novel applications such as coupling of plasmons to atomic gases are on the horizon [14].

Most of these fundamental effects as well as the early applications have been demonstrated with samples fabricated by electron-beam or ion-beam lithography. This method is quite advantageous for high-quality samples of limited size (typically of the order of 100 × 100 µm²), but it is very costly and not really suited for mass fabrication. In particular, nanostructures with small gaps over large areas, stacked nanostructures, or multiple materials aligned with respect to each other require a major effort. Also the creation of 3D chiral materials is extremely difficult with this method [15]. An alternative method for nanofabrication includes nano-imprint lithography, which requires a new large-area mask for each separate pattern. Such masks are usually prepared by electron-beam lithography. Direct laser writing by two-photon polymerization is a promising approach, since it allows for more flexibility and large areas, as well as for chiral structures [16–18]. However, this method is still quite costly. Interference lithography is large-area and low-cost, but only a limited range of well-ordered simple periodic structures are available [19]. Nanosphere lithography is large-area, low-cost, and simple, however, suffers from the drawback of clogging during evaporation [20–21].

Here, we present an overview over hole-mask colloidal nanolithography [22], which we combine with tilted angle rotation evaporation [23]. Our method is inherently large-area, low-cost (as it can be added simply to standard thermal or electron-gun evaporators), flexible (as programming of stepper motors allow for complex patterns), and capable of exact positioning of multiple materials in the sub-10 nm range over large areas. Also, stacking different materials or creation of 3D chiral structures is easy. First, we are going to describe the manufacturing process in detail, which consists of the hole-mask preparation and the subsequent tilted angle rotation evaporation, followed by a lift-off and cleaning procedure. Additionally, we are going to elaborate on how to choose the density of the disordered nanostructures on the substrate, which allows control of distance between neighboring nanostructures. This is important for potential multilayer processes, where a suitably low density in each step is required to avoid clustering. Also, for the investigation of single nanostructures, low-density preparation can be desirable. We are going to show representative SEM images of the fabricated nanostructures with geometries of different complexities. Finally, we are presenting the corresponding optical properties to demonstrate the high-quality and potential applications of our structures. In the accompanying videos, a general introduction to the process is given, as well as the individual processing steps are described. The videos can be found at the website of Beilstein TV [24–25].

## Experimental

### Hole-mask fabrication

**Figure 1:**
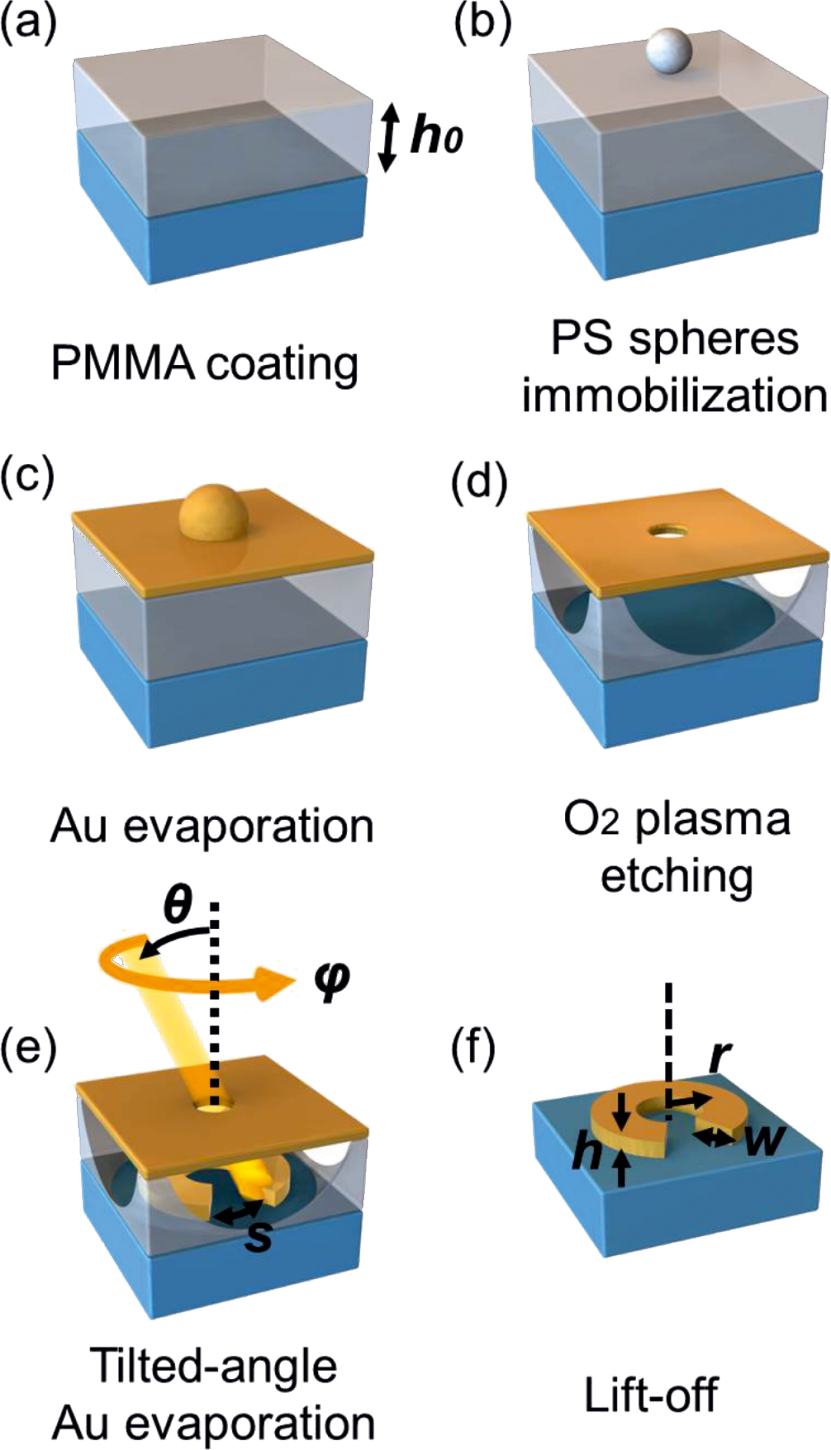
Fabrication scheme. (a) Deposition of sacrificial PMMA layer. (b) Deposition of polystyrene spheres in an arbitrarily distributed arrangement. (c) Evaporation of oxygen plasma resistant Au-mask. (d) Removing polystyrene spheres with ultrasonic bath and subsequent isotropic dry etching leads to extended holes in PMMA layer. (e) Evaporation of the structures by tilted angle evaporation (polar rotation angle φ, azimuthal tilt angle θ). (f) Lift-off of the sacrificial PMMA layer. Reproduced with permission from [23]. Copyright 2012 ACS NANO.

Before the mask fabrication, all substrates are cleaned with acetone and isopropanol in an ultrasonic bath for about 5 min, respectively, and then dried with nitrogen gas. In addition, glass substrates are functionalized directly after the cleaning process with 20 µL isobutyl(trimethoxy)silane (Sigma Aldrich) in 10 mL toluene solution for about 8 h to improve the adhesion of a polymer layer. Afterwards, a layer of poly(methylmethacrylate) (PMMA) is spin coated onto the substrate (see Figure 1a). We use different PMMA from Allresist (AR-P 661.04 and AR-P 661.06) and rotation speeds of about 4000 rpm to create 230 nm and 480 nm thick polymer layers, respectively. Varying the rotation speed enables us to tune the thickness of the PMMA layer within ±50 nm. After that, the sample is hard baked at 165 °C for 2 min to fix the PMMA layer on the substrates. For the following drop coating of the solution on the polymer film surface, we treat the sample in an oxygen plasma for about 18 seconds (Diener Pico, 0.5 mbar O_2_, power level 50% of 200 W, HF power at 2.45 GHz) to decrease the hydrophobicity of the PMMA layer. Before drop coating of the polystyrene (PS) spheres, which have negative electric surface charges, we should bring at first a net positive charge to the polymer layer for a better adherence and arrangement. Here we use a poly(diallyldimethylammonium chloride) (PDDA) solution (Sigma Aldrich, 0.2 wt %) suspendend in water. A droplet of PDDA is dripped onto the PMMA surface and the sample is immediately rinsed with deionized water and dried in a nitrogen stream.

For the preparation of the mask we use water-suspended PS spheres from Polysciences with diameters of 119 nm or 220 nm for different structures sizes. Typically, for 119 nm (220 nm) PS spheres, a 230 nm (480 nm) thick PMMA layer provides the best results. The purchased PS solution is diluted to 0.01 wt % and then ultrasonicated for about 20 min. By varying the concentration of the solution of PS spheres we can adjust the coverage of the evaporated structures. The details will be discussed below. After charging the PMMA layer with the PDDA solution, a droplet of the PS spheres solution is dripped onto the sample. After 1 min, it is rinsed away with deionized water and the sample is placed in a hot water bath at about 98 °C for around 3 min to fix the PS spheres on the polymer film. After drying the sample with nitrogen, the PMMA layer is now covered with well separated and randomly arranged PS spheres. Subsequently, a thin film consisting of 5 nm chromium (Cr) and 20 nm gold (Au) is evaporated on top of the sample (see Figure 1c), as an oxygen plasma resistant layer, and the PS spheres are removed by using deionized water and an ultrasonic bath (90 W, 20 min). Finally, we treat the sample again with the oxygen plasma for an isotropic etching to create extended holes in the PMMA layer (for 230 nm PMMA 11.2 min, power level 100% of 200 W, 1.3 mbar O_2_) underneath the holes of the gold mask (see Figure 1d). For 480 nm of PMMA, the etching time should be extended to 13.5 min. This etching step is very crucial for a successful fabrication and depends strongly on the etching parameters, which may differ for different plasma etching machines. A good indication for successful etching is a suitable waviness of the sample afterwards, due to the removed PMMA around the mask-holes underneath. Additionally, SEM imaging is helpful to determine whether the PMMA layer has been etched all the way down and a sufficient number of posts still remains to support the Au evaporation hole-mask. If this step was successful, the samples are ready for tilted angle evaporation.

### Tilted-angle-rotation evaporation

**Figure 2:**
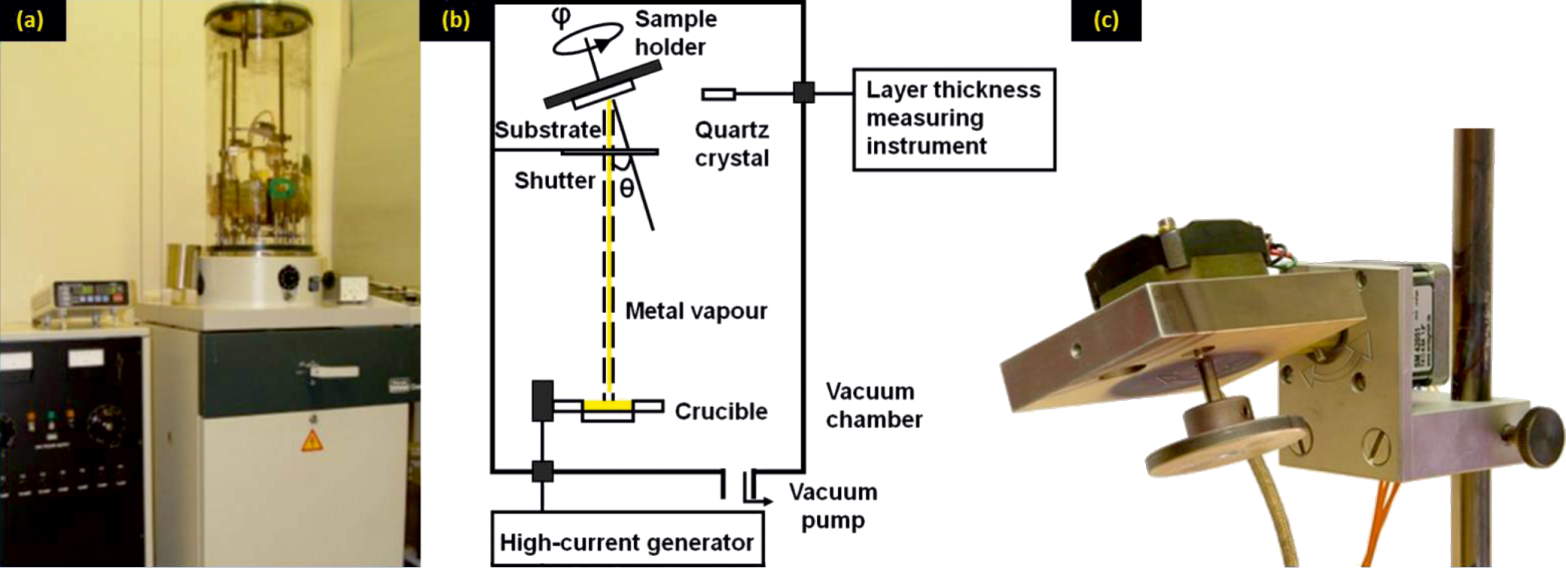
Tilted angle evaporation setup. (a) We modify an Edwards E306 evaporation machine by using a 60 cm high glass cylinder in order to allow for a larger crucible-sample distance during evaporation. This warrants a point-like and straight evaporation. (b) Schematic setup of the evaporation, with the two additional stepper motors (c) that can rotate the substrate during evaporation around a polar angle φ and an azimuthal angle θ. The electric leads are connected by a vacuum feedthrough to an external controller outside the vacuum chamber and controlled by a computer program that allows for different evaporation sequences.

We use a modified thermal evaporation machine Edwards E306 (see Figure 2a) combined with two vacuum-compatible stepper motors (see Figure 2c) inside the evaporation chamber for the evaporation process. The sample can be rotated with the two motors in azimuthal and polar directions, which is controlled with the software SMCView during the evaporation. Combined with a controllable shutter between the sample and the materials crucible, many different and complex plasmonic nanostructures can be fabricated, which will be shown later. For the standard fabrication of gold nanostructures, a thin adhesion layer of Cr is evaporated at first onto the substrates. As the amount of Cr is too small, it can be neglected in the analysis of the measured optical properties and the simulations. The mask-hole size, the azimuthal angle φ and the polar angle θ, as well as the PMMA thickness *h*_0_ determine the geometry of the structure. For example, as shown in Figure 1e and Figure 1f, the width of the SRRs *w* only depends on the mask-hole diameter, namely, the size of the used nanospheres. The average radius of the SRRs depends on the PMMA thickness *h*_0_ and the azimuthal angle θ:

[1]
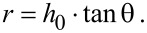


The gap size *s* depends additionally on the rotated angle φ:

[2]
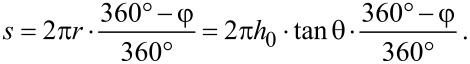


After evaporation has been carried out, the sacrificial PMMA layer and the gold mask are removed by adhesive tape. In order to eliminate residues the sample is then immersed into an acetone solution and treated in an ultrasonic bath for about 1 min. Some sensing applications, e.g., SEIRA, require further cleaning which can be realized by oxygen plasma etching (10 to 15 min).

### Details on mask preparation – variation of nanostructure density

**Figure 3:**
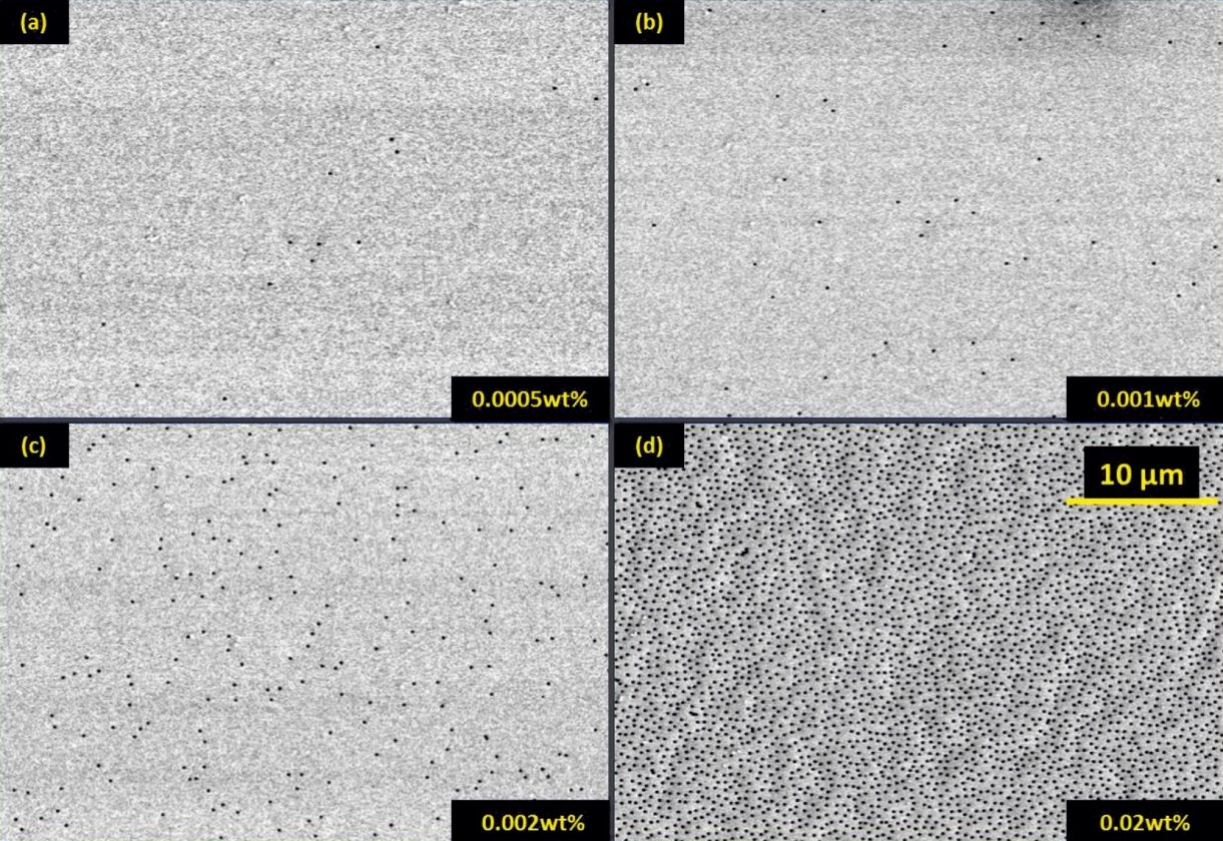
SEM images of gold masks with different concentrations of PS spheres (shown in the lower-right corners). The PDDA concentration was 0.05 wt % for (a) and (b), 0.1 wt % for (c), and 0.2 wt % for (d). The wavy structure in Figure 3d is indicative that a sufficient oxygen plasma etching time has been reached. PS nanospheres of 119 nm diameter were used in all four examples.

In order to realize different plasmonic applications, the density of the disordered drop-coated nanospheres must be well controlled by suitable concentrations of the negatively charged PS nanospheres and the positively charged PDDA solution. The electrostatic and steric forces have to be in a careful equilibrium to realize a wide range of densities of the PS spheres on the PMMA substrate after drop-coating. The following values are typical for 119 nm PS spheres, whereas other sphere diameters require different concentrations. For a low coverage with structures a low concentration of the PS solution such as 0.0005 wt % or 0.001 wt %, is required as shown in Figure 3a and Figure 3b. For the charge balance, we use 0.05 wt % PDDA for both of the PS solutions. A certain PDDA concentration is suitable for different PS concentrations within a certain range. The mask in Figure 3a shows a really low coverage of structures, which even allows for the investigation of single nanostructures.

A low coverage of structures is necessary for the multiple fabrications of multilayers. In fact, we prepare a first layer with a given structure, followed by two more fabrication sequences that consist of a new dilute mask and subsequent pattern evaporation on the same sample. The density should be tuned carefully to a suitably low value. High values will result in clustering due to the overlap of structures present in different fabrication processes. Lower values of the concentration and hence the low structure density might result in too weak an optical signal. For a slightly higher coverage we use 0.002 wt % PS solution and 0.1 wt % PDDA, as shown in Figure 3c. This recipe can be used for fabrication of three-layer structures. Many applications require pattern densities that are as high as possible without touching the neighboring structures. Therefore, a high concentration of PS spheres of about 0.02 wt % and a PDDA concentration of 0.2 wt % is required. A typical example of such a prepared hole-mask is shown in Figure 3d. Due to the electrostatic repulsion, a certain nanopattern density cannot be exceeded. The upper density limit for 119 nm diameter PS spheres is reached for a concentration of about 0.01 wt %. Increasing the concentration to 0.02 wt % or even higher values did not result in higher pattern densities. Even at highest PS concentrations we did not observe clustering. We should note that the concentration value of PDDA for the highest pattern density has also turned out to be suitable for 220 nm diameter PS spheres, which has an upper density limit for a concentration of about 0.08 wt %.

## Results and Discussion

**Figure 4:**
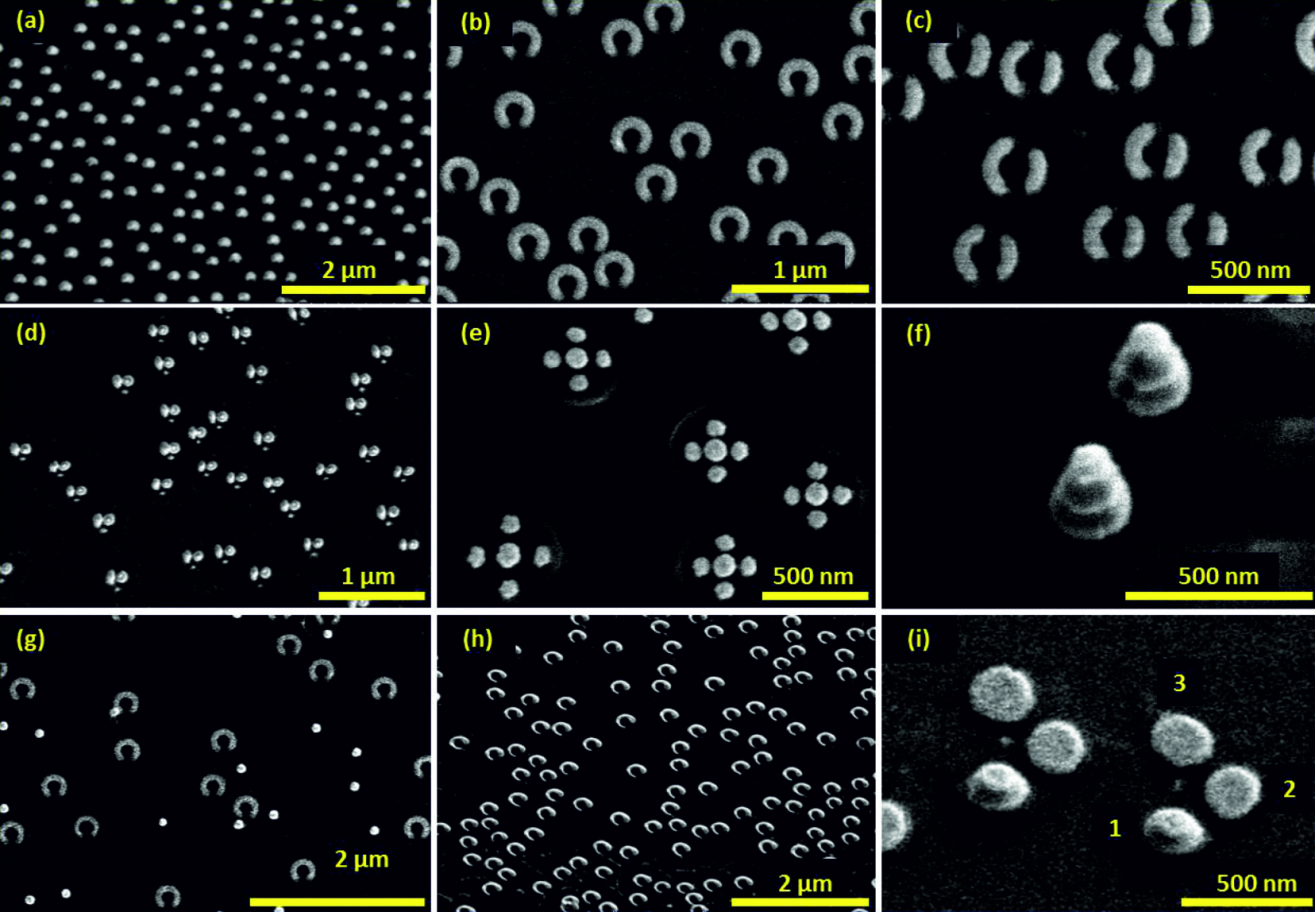
A variety of different nanostructures fabricated by hole-mask colloidal lithography: (a) ellipses, (b) split-rings, (c) asymmetric double split-rings, (d) dimers, (e) pentamers, (f) three-layer stacked gold disks, (g) disks and split-rings by multilayer fabrication, (h) 3D chiral gold spirals, (i) 3D chiral tetramers. All structures are fabricated with hole-masks, which are prepared by 119 nm diameter PS spheres and 230 nm PMMA, except samples (f) and (i), for which 220 nm diameter PS spheres and 480 nm PMMA are used. The small disks near the dimer structures in panel (d) stem from the clogging effect of the mask during the evaporation of the third structure part.

**Figure 5:**
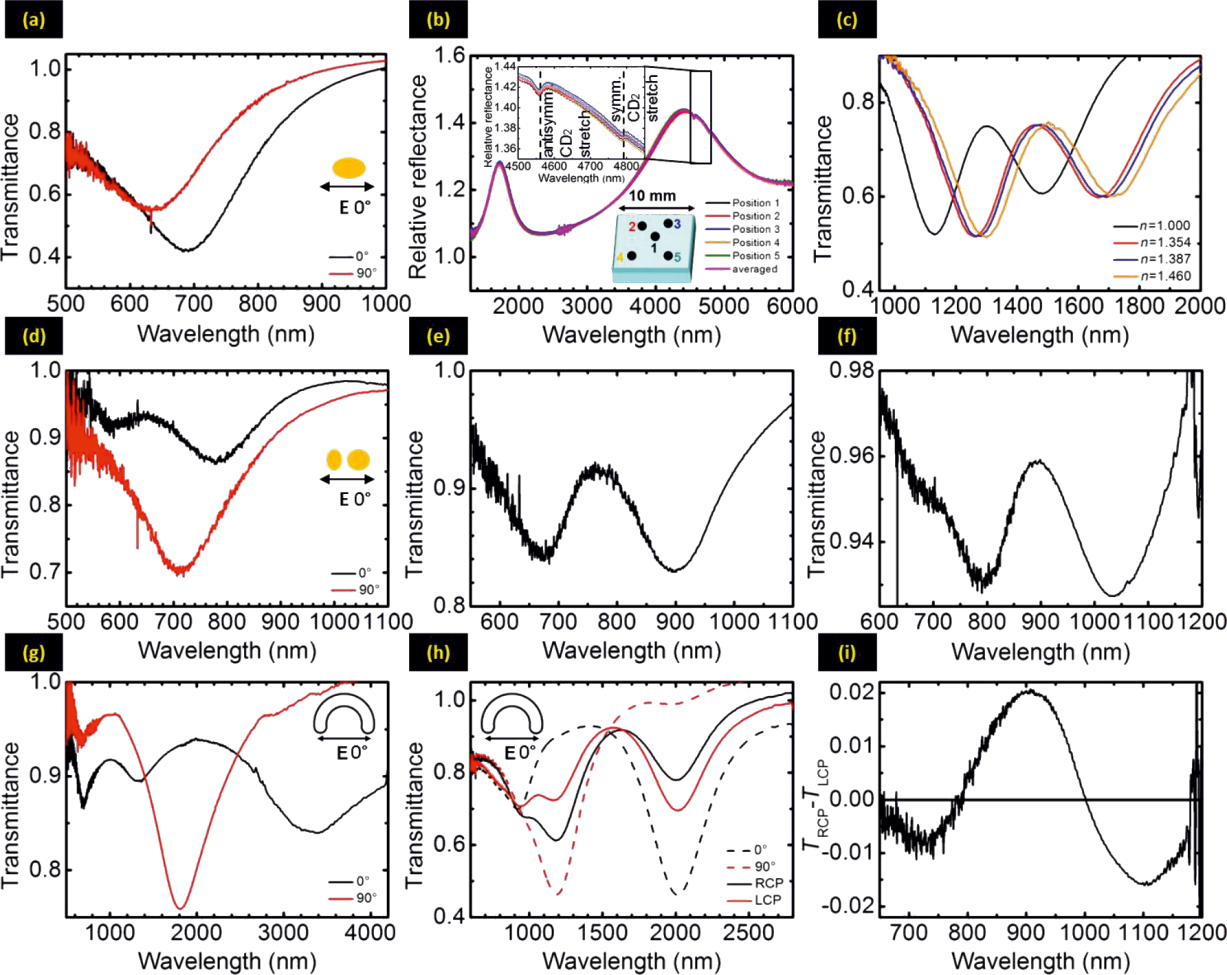
Optical spectra of the fabricated nanostructures as shown in Figure 4. The measurements are performed with a Fourier transform infrared spectrometer under normal incidence. Detailed descriptions see text.

In the following, we show a variety of plasmonic nanostructures that were fabricated by using our technique. Figure 4 shows scanning electron microscopy (SEM) images of selected nanostructures, whereas Figure 5 depicts their respective optical spectra. All structures are fabricated with hole-masks prepared by 119 nm diameter PS spheres and 230 nm PMMA, except samples (f) and (i), for which 220 nm diameter PS spheres and 480 nm PMMA are used. Only for the sample (4b) we use a silicon substrate, and for all other samples glass substrates are used.

Figure 4a shows simple elongated metallic nanostructures, which are evaporated with a small constant tilt angle of θ = 13° and a polar rotation angle of φ = 100°. The gold thickness is about *h* = 30 nm and the resulting nanostructures have dimensions of about 200 nm × 150 nm. In fact, they represent very small SRRs with a slightly curved shape. Figure 5a demonstrates the anisotropic resonances, resulting in particle plasmons around 690 and 630 nm parallel (0°) and perpendicular (90°) to the long structure axes. The concentration of PDDA was 0.2 wt % and the PS concentration was 0.02 wt %. In principle, elongated nano-antennas can be also fabricated by the variation of the angle θ around the normal incidence position.

Figure 4b shows SRR structures with average radius *r* = 100 nm, width *w* = 100 nm and thickness *h* = 20 nm, which are fabricated with an azimuthal angle of θ = 22.5° and a polar angle of φ = 288°. During the evaporation process, which takes roughly 300 s, about 55 back-and-forth rotation cycles are carried out. The rotation speed around the angle φ is approximately 108 °/s. If one uses fewer rotation cycles during evaporation, clogging of the holes in the gold mask by evaporated metal will lead to smaller widths. In contrast, more cycles enable the fabrication of a very homogeneous structure geometry. In principle, one could even utilize this clogging effect for the fabrication of tapered structures with diminishing width, in the extreme case by using just one single rotation. This SRR sample can be used for SEIRA measurements [23]. We cover the SRRs with one monolayer of perdeuterated 1-octadecanethiol (SD(CD_2_)_17_CD_3_, *d*-ODT) and present the reflectance spectra for polarization along the SRR gaps in Figure 5b. In the spectra we observe a fundamental SRR resonance at about 4.4 µm. The symmetric CD_2_ vibrational mode at 4790 nm (2089 cm^−1^) and the anti-symmetric CD_2_ vibrational mode at 4560 nm (2195 cm^−1^) of the *d*-ODT nearly coincides with the fundamental SRR mode and both of them are strongly enhanced. Additionally, we record the reflectance measurements by using a circular aperture with a diameter of about 100 µm at different positions on the sample with separation distances in the millimeter-range. As shown in Figure 5b, the SRR plasmon resonance, as well as the two vibrational modes of the *d*-ODT molecule is nearly the same for all the measurements. This experiment verifies the extremely homogeneous arrangement of the SRR structures over large-area with really low defect concentration.

Figure 4c shows asymmetric double SRRs with thicknesses of about 15 nm [26]. The radius of the ring is approximately 100 nm, which corresponds to an azimuthal angle θ of 22.5°. The rotation angles, φ, for the two arms are 170° and 130°, respectively. Both gap angles are about 30° (about 45 nm). Such double SRRs have been demonstrated to be suitable for supporting Fano resonances [4]. A plasmonic Fano resonance is defined as the coupling of a broad bright mode with a narrow dark mode, which cannot be exited directly with the incident light. It shows an asymmetric shape in the spectrum and has an extremely small resonance width. Furthermore, Fano resonances show a very high sensitivity for the change of the surrounding refractive index, which is a singularly appropriate platform for localized surface plasmon resonant (LSPR) sensing [5–8]. In order to evaluate the suitability of this first large-area low-cost Fano structures for refractive index sensing, we use this asymmetric double split-ring sample and measure the transmittance spectra (see Figure 5c) with incident linear polarization along the arc length upon exposure to ethanol (*n* = 1.354), butanol *(n* = 1.387) and glycerin (*n* = 1.460). The refractive indices are taken at about 1500 nm. We observe a well-modulated Fano resonance at about 1300 nm for *n* = 1.0 (black curve) due to the coupling of the bright superradiant and dark subradiant hybridized modes and the expected red-shift of all spectral features upon exposure to the liquids with higher refraction indeces. Additionally, we use the figure of merit (FOM) [27] to study the sensitivity of the Fano resonance quantitatively:

[3]
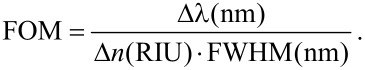


Here FWHM is the full width at half maximum of the Fano resonance and defined for the Fano resonance as the distance between the antipeak on the short wavelength side and the peak [28]. The ratio Δλ/Δ*n* is the resonance shift per refractive index unit (RIU). As shown in Figure 5c, the Fano peak shift is about 460 nm per refractive index unit in experiment and 560 nm in simulation. The FWHM is around 170 nm in measurement and 196 nm in simulation, resulting in a FOM about 2.7 and 2.9, respectively. The longer-wavelength dip in the transmittance spectra is even more sensitive than the Fano peak, which shows Δλ/Δ*n* around 520 nm/RIU in experiment and 605 nm/RIU in simulation. The corresponding FOMs are 2.9 and 4.8, respectively. In the previous research, the sensitivities of some single-particle LSPR sensors have reached up to 665 nm/RIU and FOMs of 5.4 [29–30]. The best FOM until now was up to 5.7, when electron beam lithographic structures were used [31], which usually requires expensive machines and long-time fabrication processes. Regarding our low-cost and fast method, our samples are quite suitable for mass manufacturing of large-area LSPR sensors. The quality of the samples, as well as the FOM for sensing application, could be even easily improved in the future, for example, by using thicker structures, more suitable designed geometries, and variation of evaporation conditions.

Figure 4d shows bimetallic nanostructures, consisting of an elongated gold bar at the left and a palladium disk on the right (both with a height of 20 nm) with roughly 100 nm diameter and 20 nm gap in between. The spectra in Figure 5d depict a long wavelength mode around 780 nm as well as a small peak at 580 nm for a polarization of 0°, which is along the dimer axis (black curve), and a shorter wavelength mode around 710 nm for perpendicular polarization (red curve). Possible explanations include the hybridization between the two particle plasmons in different polarizations, which results in attractive and repulsive interaction, leading to red- and blue-shifted peaks (for 0° and 90° polarization) in comparison to a single particle. The small feature at 580 nm could arise from the excitation of the antisymmetric hybridized mode with parallel polarization along the dimer axis. Another possible explanation is the presence of the Pd disk in the gold plasmon hotspot for 0° polarization and its absence close to the gold hotspot for 90° polarization. This could red-shift the gold plasmon for 0° polarization when compared with 90°.

In Figure 4e, we demonstrate the power of our method to fabricate very complex plasmonic oligomers, namely a gold pentamers. The four outer dots are created first with azimuthal tilted angle θ of 25°. Their thickness is 15 nm and their diameter is 100 nm. The tip to fabrication the same size outer ring disks is evaporation of fewer (e.g. 5 nm instead of 20 nm) materials for each one and repetition of the process in a reverse order and then in a normal order again until enough thickness of the structures is reached. This will reduce the clogging effect and achieve four outer ring disks in the same size. In a last step, the central disk is evaporated, and its diameter is about 120 nm, which is slightly larger than the outer ring disks, since the gold is evaporated normal to the substrate. Thus, the clogging effect due to the shadowing is less dramatic and still a height of 15 nm is reached. The optical transmittance in Figure 5e shows a well-modulated Fano resonance at 760 nm, visible as a transmittance maximum. This spectral shape results from the interaction between bright and dark collective modes, as explained in great detail by other authors [1,32–34].

In Figure 4f, we demonstrate the stacking capabilities of hole-mask colloidal lithography. The SEM shows a 5-layer structure, consisting of three gold layers with 20 nm thickness and two dielectric spacer layers of MgF_2_ with 70 nm thickness inbetween. The fabrication was carried out by using 220 nm diameter PS spheres and 480 nm PMMA. Due to the large total thickness of these nanostructures of 200 nm, the clogging effect is clearly visible and leads to a vertical tapering which results in a strongly decreasing structure width. The optical transmission spectrum shows three resonances, probably due to the three different gold disks of different size. The smallest disk features the shortest resonance wavelength and oscillator strength, which is present only as a shoulder at 680 nm. Hybridization effects might also influence the spectrum, as can be inferred from simulations (not shown here). At a large spacer thickness of 70 nm, strong phase retardation effects are already present, and one needs to consider far-field coupling effects [35].

Figure 4g shows an example of multishape fabrication by using hole-mask lithography. In this case, a two-shape structure consisting of gold SRRs and disks was produced on the same glass substrate by repeating the whole fabrication sequence twice. The key issue is a sufficiently low pattern density of the first layer in order to avoid overlapping patterns. As a result we obtain a SRR pattern with the same structure geometry and evaporation parameters as that shown in Figure 4b. The disks have the same diameter as the 119 nm diameter PS spheres and a thickness of about 25 nm. The transmittance spectra (Figure 5g) of this sample features the first-order SRR mode at about 3.4 µm and the third-order mode at a wavelength of about 1.4 µm for 0° polarization (black curve), which is along the gap. The second-order mode at about 1.8 µm is excited by incident light with 90° polarization (red curve). All the SRR modes of this sample are blue-shifted compared to the modes of sample (4b), due to a smaller refractive index of glass in comparison to silicon substrates. Additionally, a polarization-independent resonance at about 700 nm is clearly visible in the spectra, which is the fundamental dipole mode of the disk structures. The relatively small modulation depth is due to the low structure coverage, which can be improved by increasing the concentration of PS solution and PDDA, as discussed before. With this 2-shape sample, we can combine different applications in the IR and visible range, for example to carry out simultaneous SEIRA and SERS measurements [36].

Figure 4h depicts a 3D left-handed chiral SRR structure with different widths and thicknesses of the two opposite ends [37]. The average radius is about 100 nm and the structure width shrinks from 90 nm down to roughly 20 nm whereas the thickness decreases from 45 to 20 nm at the same time. We use an azimuthal angle θ = 22.5° and a rotation angle φ = 270°. The key technique for manufacturing such 3D chiral structures is the increasing polar rotation speed during evaporation. Only one single turn is carried out during fabrication. The effect of increasing clogging of the gold-mask holes additionally causes tapering of the structures. In principle, the combination of varying angular velocity and increasing clogging can be combined to compensate or enhance the tapering effect. Additionally, during polar rotation a simultaneous variation of the azimuthal angle is possible, which leads to spiral structures. The optical spectra of this sample are shown in Figure 5h. With linearly polarized light, the first- and third-order SRR modes are observed at about 2 µm and 1 µm for 0° polarization (black dashed curve), and the second-order mode at around 1.2 µm for 90° polarization (red dashed curve). In order to study the chiral properties of our structures, we also measure the sample with circularly polarized light (solid line). Here, left-handed circularly polarized (LCP) light is defined as its electric field-vector performing a left-hand rotation during propagation towards the sample. The red curve describes the transmission spectrum with left-handed circularly polarized light and the black curve for right-hand circularly polarized (RCP) light. As circularly polarized light can be constructed from linearly polarized light by using the prescription *x* ± *iy*, all three SRR modes are visible simultaneously in the spectra [37]. The differences between the spectra measured with LCP- and RCP-light are obvious, which confirms the chiral characteristics as well as possible elliptical birefringence, which is associated with polarization conversion of our structures [1].

Afterwards, we show another fabricated chiral structure with a 3D oligomer shape, which consists of three gold disks in the first layer that are arranged in an L-shaped pattern, followed by a spacer and another disk above the first disk, as shown in Figure 4i. For this sample we use 220 nm diameter PS spheres and 480 nm PMMA. The azimuthal tilted angle θ is 21°, and we use MgF_2_ as spacer between the stacked two gold disks. The gold and MgF_2_ thicknesses are about 20 nm and 35 nm, respectively. The upper layer disks are slightly smaller due to the clogging effect. In order to study the chiral optical properties of this pattern, we introduce at first circular dichroism (CD). Circular dichroism is defined as the difference in absorbance for right- and left-handed circularly polarized light. Our experimental-setup only allows reflectance and transmittance measurement for circularly polarized light in a very small frequency range. Within this spectral region the differences between absorbance and extinction (1 − transmittance) indicate no significant deviations [38]. Therefore, we calculate the transmittance difference (Δ*T*) between RCP- and LCP-light to determine the chiral properties of our sample (Figure 4i), and plot them in Figure 5i. Due to the same orientation of all structures, which exhibits no *C*_3_ or *C*_4_ symmetry of the arrangement, the elliptic birefringence arises from the biaxiality of our sample. Therefore, contributions of polarization conversion to the measured Δ*T*_RCP-LCP_ spectra are expected. According to this, we use the calculation introduced in references [1,17] to obtain the real chiral response:

[4]
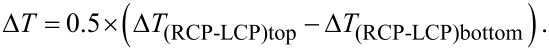


We measure the transmittance difference Δ*T*_RCP-LCP_ of our sample with both top and bottom illumination, and assume that the mean value determines correct Δ*T*-values for CD. The spectrum clearly shows non-zero values, which signifies a strong plasmonic chiral response. The detailed discussion of the relationship between the sign of the CD spectra and the handedness of the related modes is discussed in [37,39]. Such structures might be very useful in the future for the generation of local chiral fields for sensing.

## Conclusion

In this paper, we demonstrated the flexibility of hole-mask colloidal nanolithography in combination with tilted-angle-rotation evaporation to fabricate large-area complex plasmonic nanostructures of different levels of complexity at low costs. The lower limit of the fabricated structure size is about 80 nm, due to the experimental limitation. The upper limit depends on the used PS sphere and PMMA system. Small reproducible nanometer gaps over large areas, multiple materials, and 3D chiral structures are specifically well suited for our method. Hybrid nanostructures that are directly deposited on or under functional surfaces such as magneto-optical layers, organic LEDs or solar cells have been achieved. Our fabrication requires only a spin-coater, a hot plate, an oxygen plasma asher, and an evaporator which is modified with computer-controlled stepper motors. (The parts are available as a complete kit from the authors including software with the structures presented in this paper, as well as a demo DVD and a manual.) We described different simple and hybrid nanostructures and metamaterials and depicted their high-quality optical spectra. In the future, this method can be expanded to multishape fabrication processes, which, in combination with different materials and stacking, can give the most complex nanostructures that would be very hard to manufacture with any other method, in particular at such large areas and low costs.
